# Frankincense oil derived from *Boswellia carteri *induces tumor cell specific cytotoxicity

**DOI:** 10.1186/1472-6882-9-6

**Published:** 2009-03-18

**Authors:** Mark Barton Frank, Qing Yang, Jeanette Osban, Joseph T Azzarello, Marcia R Saban, Ricardo Saban, Richard A Ashley, Jan C Welter, Kar-Ming Fung, Hsueh-Kung Lin

**Affiliations:** 1 Arthritis and Immunology Research Program, Oklahoma Medical Research Foundation Microarray Research Facility, Oklahoma City, OK 73104, USA; 2 Department of Urology, University of Oklahoma Health Sciences Center, Oklahoma City, OK 73104, USA; 3 Department of Physiology, University of Oklahoma Health Sciences Center, Oklahoma City, OK 73104, USA; 4 Department of Comparative Medicine, University of Oklahoma Health Sciences Center, Oklahoma City, OK 73104, USA; 5 Department of Pathology, University of Oklahoma Health Sciences Center, Oklahoma City, OK 73104, USA; 6 Oklahoma City Veterans Affairs Medical Center, Oklahoma City, OK 73104, USA

## Abstract

**Background:**

Originating from Africa, India, and the Middle East, frankincense oil has been important both socially and economically as an ingredient in incense and perfumes for thousands of years. Frankincense oil is prepared from aromatic hardened gum resins obtained by tapping *Boswellia *trees. One of the main components of frankincense oil is boswellic acid, a component known to have anti-neoplastic properties. The goal of this study was to evaluate frankincense oil for its anti-tumor activity and signaling pathways in bladder cancer cells.

**Methods:**

Frankincense oil-induced cell viability was investigated in human bladder cancer J82 cells and immortalized normal bladder urothelial UROtsa cells. Temporal regulation of frankincense oil-activated gene expression in bladder cancer cells was identified by microarray and bioinformatics analysis.

**Results:**

Within a range of concentration, frankincense oil suppressed cell viability in bladder transitional carcinoma J82 cells but not in UROtsa cells. Comprehensive gene expression analysis confirmed that frankincense oil activates genes that are responsible for cell cycle arrest, cell growth suppression, and apoptosis in J82 cells. However, frankincense oil-induced cell death in J82 cells did not result in DNA fragmentation, a hallmark of apoptosis.

**Conclusion:**

Frankincense oil appears to distinguish cancerous from normal bladder cells and suppress cancer cell viability. Microarray and bioinformatics analysis proposed multiple pathways that can be activated by frankincense oil to induce bladder cancer cell death. Frankincense oil might represent an alternative intravesical agent for bladder cancer treatment.

## Background

Frankincense resin is obtained from trees of the genus *Boswellia *(family Burseraceae). Incisions are made in the trunks of the trees to produce exuded gum, which appears as milk like resin. The resin hardens into orange-brown gum resin known as frankincense. There are numerous species and varieties of frankincense trees, including *Boswellia serrata *in India, *Boswellia carteri *in East Africa and China, *Boswellia frereana *in Somalia, and *Boswellia sacra *in Arabia, each producing a slightly different type of resin. Differences in soil and climate create more diversity in the resins, even within the same species. The aroma from these resins is valued for its presumed healing properties and superior qualities for religious rituals since the time of the ancient Egyptians [1], and has been used in incense, fumigants, and as a fixative in perfumes.

Frankincense resin has been considered throughout the ages to have a wealth of health supporting properties. The resins of *Boswellia carteri *and *Boswellia serrata *have been used for the treatment of rheumatoid arthritis and other inflammatory diseases [2] such as Crohn's disease [3] in traditional medicine of many countries. The anti-inflammatory activity has been attribute to the resin's ability in regulating immune cytokines production [4] and leukocyte infiltration [5,6]. *Boswellia serrata *extract also exhibits anti-bacterial and anti-fungal activities [7]. Additionally, extracts from *Boswellia *species gum resins might possess anti-cancer activities, based on their anti-proliferative and pro-apoptotic activities in rat astrocytoma cell lines [8] and in human leukemia cell lines [9], as well as their anti-carcinogenic activity in chemically induced mouse skin cancer models [10]. Clinically, extract from the resin reduces the peritumoral edema in glioblastoma patients [8] and reverses multiple brain metastases in a breast cancer patient [11]. These results suggest that frankincense resin contains active ingredients that modulate important biological activities.

In search of the active medicinal ingredients of frankincense resins, Chevrier *et al*. reported that ethanol extract of *Boswellia carteri *resin comprises 7 boswellic acids [4]. Akihisa *et al*. reported that methanol extract of *Boswellia carteri *resin consists of 15 triterpene acids, including boswellic acids, and 2 cembrane-type diterpenes [12]. 11-keto-β-boswellic acid, the most potent anti-inflammatory component of the resin, selectively blocks leukotriene biosynthesis through inhibiting 5-lipoxygenase activity in rat neutrophilic granulocytes [13] and provides protective effects in a chemically induced mouse ulcerative colitis model [14]. Boswellic acids also prevent endotoxin/galactosamine-induced hepatitis in mice [15]. In addition, boswellic acids have been shown to possess anti-cancer activities through their cytostatic and apoptotic effects in multiple human cancer cell lines including meningioma cells [16], leukemia cells [17], hepatoma cells [18], melanoma cells, fibrosarcoma cells [19], and colon cancer cells [20].

Frankincense oil, an extract prepared by steam distillation from frankincense gum resin, is one of the most commonly used oils in aromatherapy practices. There has been considerable work done on the composition of frankincense oil from different species and commercial brands; and the constituents of frankincense oil differ according to the climate, harvest conditions, and geographical sources of frankincense resins [21]. Due to the contribution of boswellic acids, it is possible that frankincense oil also holds anti-cancer and anti-neoplastic properties. In this study, we demonstrated that a commercial source of frankincense oil can discriminate bladder cancer J82 cells from normal bladder urothelial UROtsa cells and suppress cancer cell viability. Based on gene expression analysis, frankincense oil activated several anti-proliferative and pro-apoptotic pathways that might be responsible for frankincense oil-induced cell death in J82 cells.

## Methods

### Reagents and chemicals

Cell culture medium [MEM and DMEM/F-12 (1:1)], fetal bovine serum (FBS), MEM vitamin solution, non-essential amino acids, epidermal growth factor (EGF), insulin-transferrin-sodium selenite (ITS) media supplement, sodium pyruvate, and penicillin-streptomycin were purchased from Invitrogen (Grand Island, NY). Frankincense oil containing 1,200 mg/ml frankincense gum resin was obtained from Young Living Essential Oils (Lehi, UT). XTT cell proliferation assay and *in situ *cell death detection kits were obtained from Roche (Indianapolis, IN). Trypan blue was purchased from Sigma (St. Louis, MO). RNeasy^® ^Mini Kit was obtained from Qiagen (Valencia, CA).

### Human bladder cell lines

Bladder transitional cell carcinoma J82 was obtained from ATCC (HTB-1; Manassas, VA). The J82 cell line was derived from a poorly differentiated, invasive human transitional cell bladder carcinoma (stage 3) [22]. J82 cells were maintained in growth medium consisting of MEM supplemented with 10% FBS, 0.1 mM non-essential amino acids, 1 mM sodium pyruvate, 2% MEM vitamin solution, 100 units/ml penicillin, and 100 μg/ml streptomycin. The UROtsa cell line was originally isolated from a primary culture of normal human urothelium and immortalized with a construct containing the SV40 large T antigen [23]. UROtsa cells were cultured in DMEM/F12 supplemented with 10 ng/ml EGF, 1× ITS media supplement, 100 units/ml penicillin, and 100 μg/ml streptomycin. Cells were maintained in a humidified cell incubator at 37°C and 5% CO_2 _and passaged every 3–4 days or when cells reached about 80% confluence.

### Cell viability analysis

To determine number of viable cells following frankincense oil treatment, J82 and UROtsa cells were seeded in 96-well tissue culture plates at the density of 1 × 10^4 ^cells/mm^2 ^in 100 μl growth medium. Following overnight incubation for adherence, 100 μl cell growth media or varying dilutions of frankincense oil (at1:600 to 1:4,000 final concentration) in their growth media were added to each well in triplicate to make a total of 200 μl. Cell viability was determined at the time of treatment and at 24 hours following frankincense oil exposure using the XTT cell proliferation assay kit. Briefly, at the time of assay, 100 μl growth media were removed from each well, and an aliquot of 50 μl XTT labeling mixture was added back to each well. Reactions were performed at 37°C for 4 hours. Absorbance was obtained by reading the plates at 450 nm wavelength using μQuant microplate reader (Bio-Tek; Winooski, VT). Absorbance values obtained at 24 hours for untreated and frankincense oil-treated cells were normalized to the values obtained at the time of treatment to calculate fold changes in cell survival.

Trypan blue exclusion was also included to determine cell viability following frankincense oil treatment. Briefly, J82 and UROtsa cells were seeded in 24-well tissue culture plates at the same density as used in XTT assay in 500 μl growth media. Following adherence, cells received either 500 μl of growth medium or varying dilutions of frankincense oil in each well. At 3 hours after frankincense oil treatment, the culture medium was collected to save the non-adherent cells; and the remaining cells were trypsinized and combined with the cells harvested from the culture medium. The cells were collected by centrifugation, and re-suspended with 200 μl phosphate buffered saline (PBS). Then, an aliquot (20 μl) of the cell suspension was mixed with the same volume of 0.4% (w/v) trypan blue solution. The cells were counted using a hemocytometer to determine the numbers of blue cells (non-viable) and bright cells (viable). Cell viability was expressed as the percentage of trypan blue positive cells compared to the total number of cells.

### RNA extraction and quality evaluation

Total RNA was isolated from J82 cells for microarray analysis. Briefly, 2 × 10^5 ^J82 cells were seeded in 60 mm tissue culture plates, cultured overnight for adherence, and either left untreated or treated with 1:1,000 dilutions of frankincense oil in growth medium. Total RNA was isolated at 0 hours (no treatment) and at 0.5, 1, 2, and 3 hours after stimulation using the RNeasy^® ^Mini total RNA isolation kit based on manufacture's recommendations (Qiagen; Valencia, CA). Total RNA concentration was determined with a nanodrop scanning spectrophotometer, and then qualitatively assessed for degradation using the ratio of 28:18s rRNA by a capillary gel electrophoresis system (Agilent 2100 Bioanalyzer, Agilent Technologies; Santa Clara CA).

### RNA labeling, microarray hybridization, and scanning

A total of 250 ng of RNA from each time point was labeled using the Illumina Total Prep RNA Amplification Kit following manufacturer's directions (Ambion; Austin. TX). Briefly, cDNA was reverse transcribed from RNA after priming with T7-oligo-dT, and cRNA was synthesized *in vitro *from the T7 promoter while incorporating biotinylated UTP. cRNA was hybridized overnight to Illumina human Ref-8 version 3 BeadChips containing probes for a total of 24,526 transcripts. Microarray chips were washed to high stringency and labeled with streptavidin -Cy3 (Amersham Biosciences; Piscataway, NJ) prior to scanning on an Illumina BeadArray Reader.

### Bioinformatics data analysis

Non-normalized fluorescent intensity of each probe on the microarray slide was obtained using the DirectHyb gene expression package in BeadStudio software (Illumina, version 3.1.3). Fluorescent intensity filtering was performed to remove genes that lacked a minimum relative fluorescence of 64 units in at least one time point. Data from the remaining probes were log transformed and quantile normalized (Matlab). A final filtering was performed to identify genes with a minimum two-fold change in normalized expression values between adjacent time points. Expression data are available on Gene Expression Omnibus (GEO) with accession number GSE14002.

### TUNEL (terminal deoxynucleotidyl transferase dUTP nick end labeling) Analysis

TUNEL analysis was performed in J82 cells using an immunohistochemical (IHC)-like staining procedure as we reported [24]. Briefly, adherent J83 cells were either left untreated or treated with 1:1,000 dilution of frankincense oil. At 3 hours after treatment, both non-adherent and adherent and cells were collected following centrifugation. Cell pellets were fixed in 10% formalin, immersed in 2% agarose, and subjected to paraffin embedding. The embedded cell blocks were sectioned, dewaxed, and rehydrated. Apoptotic cells were detected using the *in situ *cell death detection kit. Following the terminal deoxynucleotidyl transferase reaction, fast red substrate was added for color development. Slides were then washed and sealed with an aqueous mounting medium.

### DNA fragmentation analysis

To determine whether J82 cells undergo DNA fragmentation following frankincense oil treatment, 2 × 10^5 ^J82 and UROtsa cells were seeded in 60 mm tissue culture plates in their growth media, incubated overnight for adherence, and treated with a 1:1,000 dilution of frankincense oil in growth media. Cells were harvested at 0 (untreated control), 1, 3, and 6 hours following treatment; and genomic DNA was prepared and precipitated based on reported procedures [25]. Quantities of the genomic DNA were determined spectrophotometrically. Aliquots (10 μg) of the genomic DNA were separated on a 2% agarose gel; images of ethidium bromide stained gels were captured by the Gel Doc 100 system (Bio-Rad, Hercules, CA).

### Statistics

The results are expressed as mean ± SEM from four experiments. Comparisons of J82 and UROtsa cell survival following frankincense oil treatment were made using the one-way analysis of variance (ANOVA) followed by post hoc Dunnett's test. *P *< 0.05 was considered statistically significant.

## Results

### Frankincense oil-suppressed bladder cell viability

**Figure 1:**
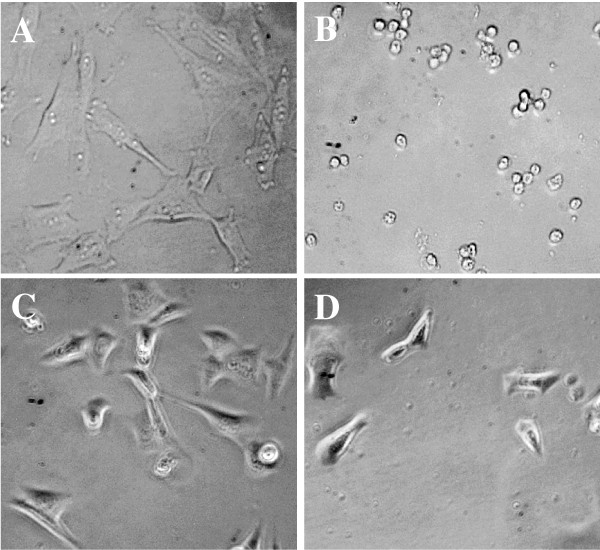
**Morphological changes of bladder carcinoma J82 and bladder urothelial UROtsa cells following frankincense oil stimulation**. Bladder J82 and UROtsa cells were seeded in 96-well tissue culture plates at the concentration of 1 × 10^4 ^cells/mm^2^, cultured overnight for adherence, and either left untreated or subjected 1:1,000 dilution of frankincense oil stimulation. Images were taken at 24 hours following treatments for (A) untreated J82 cells, (B) J82 cells treated with frankincense oil, (C) untreated UROtsa cells, and (D) UROtsa cells treated with frankincense oil using Olympus IX51 inverted microscope. Notice cell shrinkage observed in J82 cells following frankincense oil treatment. In contrast, UROtsa cells did not experience noticeable morphological alteration following the same concentration of frankincense oil exposure.

The bladder carcinoma J82 cells presented a density-independent growth and grew in soft agar, but were not tumorigenic in nude mice [26]. The immortalized bladder urothelial UROtsa cells expressed SV40 large T antigen, but did not acquire characteristics of neoplastic transformation, including growth in soft agar or the development of tumors in nude mice [23]. To determine if frankincense oil suppresses bladder cell viability, both J82 and UROtsa cells were subjected to morphological evaluation and cell viability assessment. J82 cells underwent significant morphological changes, such as detaching from tissue culture plates and shrinking beginning within 3 hours following frankincense oil exposure. At 24 hours after treatment, J82 cells completely detached from tissue culture plates whereas untreated controls remained adherent to the plates (Figure 1A and 1B). In contrast, UROtsa cells remained attached to the bottom of plates and did not show noticeable morphological alterations (Figure 1C and 1D).

**Figure 2:**
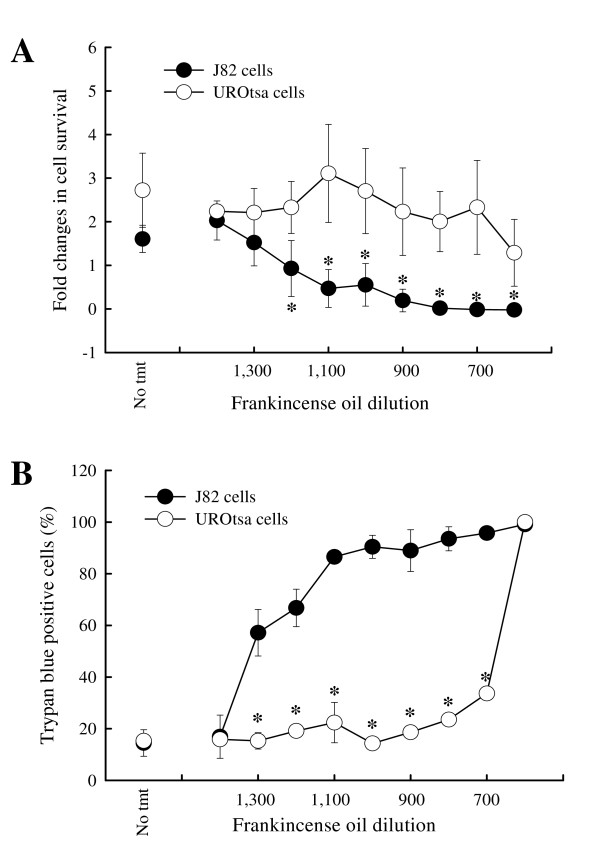
**Bladder cell survival in response to frankincense oil exposure**. Cell viability was determined using (A) a colometric XTT assay at 24 hours and (B) trypan blue exclusion at 3 hours after frankincense oil stimulation. All experiments were prepared in triplicate for XTT assay and duplicate for trypan blue exclusion. Data were presented as mean ± standard error of mean (SEM) from at least 3 independent experiments. * indicates statistical difference between frankincense oil-treated J82 cells and UROtsa cells (*P *< 0.05).

To determine whether frankincense oil affects J82 and UROtsa cell viability, the number of viable J82 and UROtsa cells was determined following various dilutions (1:600 to 1:1,400) of frankincense oil exposure. In untreated controls, number of viable J82 cells and UROtsa cells increased 1.62 ± 0.31 and 2.72 ± 0.85 fold at 24 hours following cell seeding, respectively (Figure 2). Both J82 and UROtsa cells responded to frankincense oil treatment in a dose-dependent manner. J82 cell viability decreased when cells were treated with increasing concentrations of frankincense oil. No viable J82 cells remained at 24 hours after treatment with 1:1,100 dilution of frankincense oil (0.47 ± 0.43). In contrast, UROtsa cell viability was not significantly affected by the increasing concentrations of frankincense oil until 1:600 dilution was applied to the cells. When UROtsa cells were treated with 1:600 dilution of frankincense oil, cell viability decreased to 1.29 ± 0.77 fold as compared to untreated cells. No viable UROtsa cells were detected when the concentration of frankincense oil concentration increased to 1:400 (data not shown). Based on the XTT assay, IC_50 _values (the 50% inhibitory concentrations of frankincense oil) for J82 and UROtsa cells were 1:600 and 1:1,250, respectively. Trypan blue exclusion produced results similar to the XTT assay, except that J82 and UROtsa cells seem to be more sensitive to frankincense oil treatment at 1:1,300 and 1:600 dilutions, respectively (Figure 2B).

### Identification of frankincense oil-activated gene expression

To determine the nature of J82 cell death, microarray analysis was performed. Of the 24,526 gene probes on the microarray, 8,430 probes had a fluorescent intensity value of at least twice the background intensity for one or more time points under evaluation. A total of 122 genes in J82 cells were increased above two-fold in at least two adjacent time points by frankincense oil (see Additional file 1). Only 3 of these genes increased within the first 30 min [zinc finger protein 57, the small nucleolar RNA C/D box 48, and early growth response 1 gene (EGR1)]. Levels of EGR1 mRNA increased 5.87-fold within the first 30 min, and another 2.86-fold over the next 30 min. Another 15 genes increased at least two-fold between 30 and 60 min after frankincense oil stimulation; and 11 of which continued to show elevated expression beyond the first hour. A much larger number of genes increased in expression between 1 and 2 and between 2 and 3 hours following frankincense oil exposure.

A total of 47 genes were down-regulated in J82 cells by frankincense oil (see Additional file 2). Three genes [tubulin gamma 1, vacuolar protein sorting 11 homolog, and RNA polymerase II (DNA directed) polypeptide K] were the first to decrease greater than two-fold between 30 and 60 min after frankincense exposure. Another 12 genes decreased between 1 and 2 hours, and 32 other genes decreased between 2 and 3 hours. An additional 12 genes were identified whose levels of expression changed at least two-fold between two adjacent time points and then changed in the opposite direction at least two-fold between the next adjacent time points. These 12 genes were ankyrin repeat domain 27, chromosome 5 open reading frame 34, calcineurin binding protein 1, dodecenoyl-coenzyme A delta isomerase, dynein (axonemal, intermediate polypeptide 2), ATG2 [autophagy related 2 homolog A, N-deacetylase/N-sulfotransferase (heparan glucosaminyl) 2], oviductal glycoprotein 1, plexin A3, somatostatin receptor 1, transcriptional variant 1 of rinucleotide repeat containing 5, and zinc finger and BTB domain containing 11.

### Functional grouping of frankincense oil-regulated genes

The gene products that were altered in frankincense oil-treated bladder carcinoma J82 cells were functionally grouped according to Gene Ontology classification. Based on the biological functions, gene products that function as cytokines, membrane receptors, enzymes (including kinases, peptidases, and phosphatases), and molecular transport were identified and listed in Table 1. A complete list of genes under each classification is provided in Additional file 3. Frankincense oil-regulated gene products that function as transcription factors, cell cycle arrest and cell proliferation, as well as apoptotic factors showed that frankincense oil induces cell cycle arrest and apoptosis in J82 cells.

**Table 1:** Functional groups of frankincense oil-regulated genes in bladder cancer J82 cells

**Function**	**Gene Symbol**	**Description**
Cytokines		
	CCL2	chemokine (C-C motif) ligand 2
	CCL5	chemokine (C-C motif) ligand 5
	CMTM8	CKLF-like MARVEL transmembrane domain containing 8
	CXCL2	chemokine (C-X-C motif) ligand 2
	IL1A	interleukin 1, alpha
	IL6	interleukin 6 (interferon, beta 2)
	IL8	interleukin 8
Enzymes – kinases		
	ABL2	v-abl Abelson murine leukemia viral oncogene homolog 2 (arg, Abelson-related gene)
	AXL	AXL receptor tyrosine kinase
	CDKN1A	cyclin-dependent kinase inhibitor 1A (p21, Cip1)
	CLK1	CDC-like kinase 1
	DLG1	discs, large homolog 1 (Drosophila)
	FGFR1	fibroblast growth factor receptor 1 (fms-related tyrosine kinase 2, Pfeiffer syndrome)
	PSTK	phosphoseryl-tRNA kinase
	SGK1	serum/glucocorticoid regulated kinase 1
	SNF1LK	SNF1-like kinase
	TAOK1	TAO kinase 1
	TRIB1	tribbles homolog 1 (Drosophila)
Enzymes – peptidases		
	RCE1	RCE1 homolog, prenyl protein peptidase (S. cerevisiae)
Enzymes – phosphatases		
	DUSP10	dual specificity phosphatase 10
	DUSP2	dual specificity phosphatase 2
	DUSP5	dual specificity phosphatase 5
	MTMR6	myotubularin related protein 6
	NUDT2	nudix (nucleoside diphosphate linked moiety X)-type motif 2
	PPP3R1	protein phosphatase 3 (formerly 2B), regulatory subunit B, alpha isoform
	PTPN23	protein tyrosine phosphatase, non-receptor type 23
Membrane Receptors		
	PLAUR	plasminogen activator, urokinase receptor
	PLXNA1	plexin A1
	PLXNA3	plexin A3
	SSTR1	somatostatin receptor 1

#### Transcription regulators

Two transcription factors, LOC12629 and EGR1, were immediately (within 30 min) up-regulated by frankincense oil (Table 2). Another 5 transcription factors, including ATF3, FOS, FOSB, KLF2, and ZNF234 were up-regulated within 1 hour and sustained for at least 2 hours following frankincense oil treatment. Three transcription factors, KLF4, KLF5, and ZBTB11 were up-regulated by frankincense oil between 1 and 2 hours post-treatment. The remaining 11 transcription factors (DDIT3, DEDD2, DENR, HES1, ID1, JUN, JUNB, SNAPC1, TSC22T1, UBTF, ZFP36) were considered to be late responders because their expression was altered after 2 hours of frankincense oil exposure.

**Table 2:** Frankincense oil-regulated transcription factors in J82 cells

	Time after frankincense oil stimulation (hours)			
	
	<0.5	0.5–1	1–2	2–3
Up-regulated	LOC126295 (NM_173480.1) *	ATF3 (NM_001040619.1)	KLF4 (NM_004235.3)	DDIT3 (NM_004083.4)
	EGR1 (NM_001964.2)	FOS (NM_005252.2)	KLF5 (NM_001730.3)	DEDD2 (NM_133328.2)
		FOSB (NM_006732.1)	ZBTB11 (NM_014415.1)	DENR (NM_003677.3)
		KLF2 (NM_016270.2)		HES1 (NM_005524.2)
		ZNF234 (NM_006630.1)		ID1 (NM_181353.1)
				JUN (NM_002228.3)
				JUNB (NM_002229.2)
				SNAPC1 (NM_003082.2)
				TSC22D1 (NM_006022.2)
				UBTF (NM_014233.1)
				ZNF682 (NM_033196.1)

Down-regulated	POLR2K (NM_005034.3)	ING4 (NM_198287.1)		HDAC4 (NM_006037.2)
				RAI1 (NM_030665.3)
				TAF15 (NM_003487.2)

*GenBank accession number.

#### Cell cycle arrest and cell proliferation

Several gene products identified as frankincense oil responsive genes were negatively associated with regulation of cell proliferation and positively associated with cell cycle arrest (Table 3). Genes that have been identified as anti-proliferative genes, including IL8, CLK1, DLG1, KLF4, NEDD9, CDKN1A, IL1A, IL6, and SNFILK were up-regulated in frankincense oil-treated J82 cells. In addition, DDIT3 along with IL8 and CDKNIA being identified to be responsible for cell cycle arrest were also up-regulated by frankincense oil. In contrast, H2AFX and HDAC4, genes that are responsible for DNA repair and cell cycle progression, were suppressed by frankincense oil. Other anti-proliferative genes, including SSTR1, IL1A, and IL6, were also up-regulated between 0.5 and 2 hours upon frankincense oil stimulation.

**Table 3:** Frankincense oil-regulated growth inhibitory genes in J82 cells

	Time after frankincense oil exposure(hours)				
	
Gene Symbol	0	<0.5	0.5–1	1–2	2–3
IL8	26.3	**32.6**	**146.2**	**693.6**	1241.4
CLK1	42.3	56.1	**57.9**	**121.2**	203.3
DLG1	48.3	41.2	**31.2**	**71.8**	37.2
H2AFX	105.6	103.3	**106.6**	**51.0**	96.1
ING4	73.7	63.0	**75.3**	**37.2**	43.6
KLF4	89.2	68.8	**123.2**	**326.0**	556.9
NEDD9	38.3	34.3	**23.8**	**59.7**	56.7
SSTR1	47.4	37.2	**28.4**	**70.3**	27.2
CDKN1A	56.3	54.5	95.7	**167.4**	**382.9**
DDIT3	346.7	316.0	523.2	**735.5**	**1647.7**
HDAC4	64.4	61.7	52.1	**68.0**	**31.5**
IL1A	63.4	44.5	72.1	**124.5**	**252.5**
IL6	190.5	235.4	337.2	**563.8**	**1326.4**
SNF1LK	40.6	38.8	48.9	**95.8**	**286.3**
IL8	26.3	32.6	146.2	**693.6**	**1241.4**
CLK1	42.3	56.1	57.9	**121.2**	**203.3**
DLG1	48.3	41.2	31.2	**71.8**	**37.2**

Normalized values of fluorescent intensities are presented. Bold font indicates a minimum two-fold change between adjacent time points.

#### Apoptosis

**Figure 3:**
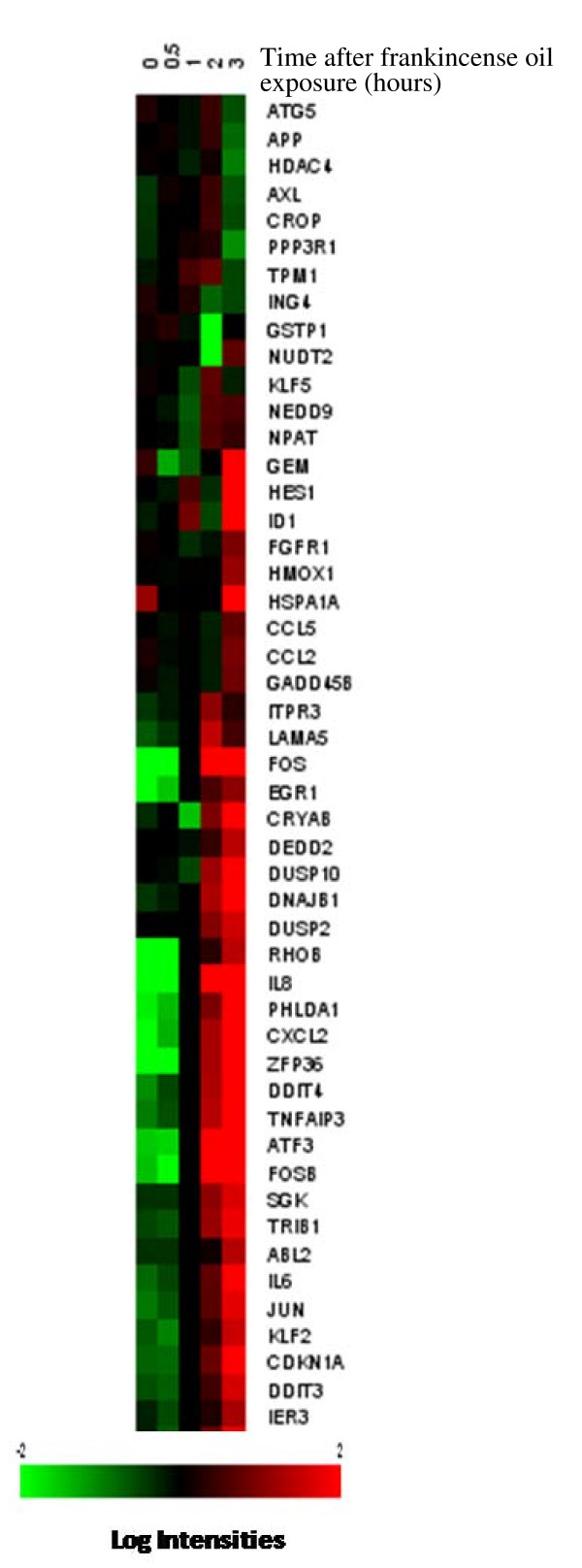
**Hierarchical clustering of frankincense oil-regulated apoptosis-related genes in J82 cells**. The map was obtained using Biometric Research Branch (BRB) ArrayTools version 3.4.0 – Beta_2 software  after log_2 _transformation of fluorescence intensities. Each column represents time intervals following frankincense oil exposure, and each row represents a gene probe set. The expression levels for individual genes are indicated by green/red color indicating an elevated/suppressed level of expression, respectively.

Levels of a large number of genes that are responsible for apoptosis were found to be modulated by frankincense oil (Figure 3). These genes included CDKN1A, DEDD2, IER3, IL6, SGK, and TNFAIP3 (up-regulated between 1 and 2 hours and remained up-regulated) as well as GAD45B, NUDT2, and others (up-regulated between 2 and 3 hours). In addition, the cell survival gene, AXL, was down-regulated by frankincense oil. However, two anti-apoptotic genes, GSTP1 and IL1A, were up-regulated. A similar contradiction was seen with a pro-apoptotic gene, ING4, being down-regulated.

### Frankincense oil-induced cell death

**Figure 4:**
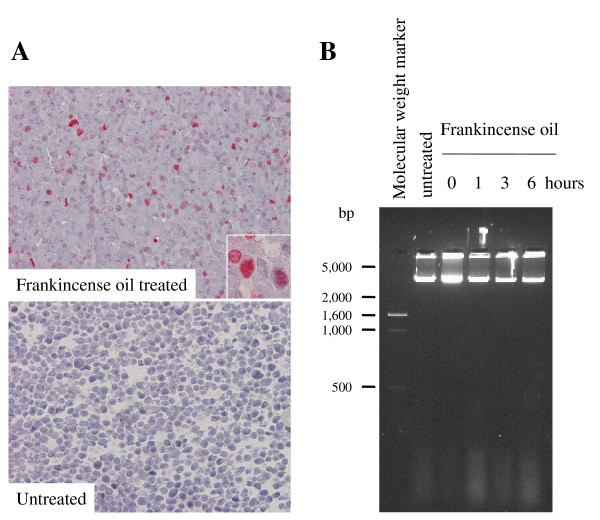
**Frankincense oil-induced J82 cell death**. To determine whether frankincense oil-induced apoptosis in bladder cancer cells, J82 cells were seeded in 60 mm tissue culture plates at the concentration of 2 × 10^5 ^cells per plate, cultured overnight for adherence, and either left untreated or treated with 1:1,000 dilution of frankincense oil. (A) TUNEL analysis was performed at 3 hours following treatment. Apoptotic cells with damaged DNA were stained positive with a bright red color (inserts). (B) DNA fragmentation was determined by separating genomic DNA on a 2% agaorse gel; and the gel image was captured using Gel Doc 100 system (Bio-Rad, Hercules, CA).

TUNEL analysis was performed to determine whether frankincense oil treated J82 cells undergo apoptosis. Frankincense oil treatment resulted in an increased number of bright red colored TUNEL positive cells as compared to untreated cells (Figure 4A). Genomic DNA fragmentation was determined between hours 1 and 6 in J82 cells following frankincense oil treatment. Agarose gel electrophoresis results showed that all genomic DNA remained as large pieces of DNA without forming a small DNA ladder (Figure 4B). There was no detectable genomic DNA for J82 cells harvested at 12 hours following frankincense oil treatment (data not shown).

## Discussion

Ranging from herbs to acupuncture, alternative medicine is becoming increasingly popular for managing health-related issues. In this brief report, we described that frankincense oil, with a window of concentration, specifically suppressed cell viability in human bladder carcinoma J82 cells, but did not affect cell viability in immortalized normal urothelial UROtsa cells. Frankincense oil suppressed J82 cell viability may be attributed to the activation of growth arrest and pro-apoptotic genes. The possibility that the witnessed differences in cell survival could be due to the presence of EGF in the media was considered. Despite this concern, we expected that UROtsa cells' resistance to frankincense oil as compared to J82 cells may not result from the presence of EGF in their growth medium based on the following observations: first, a higher concentrations of EGF (i.e. 1 μg/ml) had been reported for apoptosis protection [27], second, UROtsa cells cultured in EGF-free medium were less sensitivity to frankincense oil-suppressed cell viability as compared to J82 cells, although the overall UROtsa cell viability was reduced (data not shown), and third, four other oils, including sandalwood oil (*Santalum album*), balsam fir oil (*Abies balsamea*), palo santo oil (*Bursera graveolens*), and tsuga oil (*Tsuga canadensis*) (Young Living Essential Oils), induced nearly identical cytotoxicity in both J82 and UROtsa cells (data not shown).

Commercial frankincense oil was directly applied in our experimental cell models without modification. It was not our intention in this preliminary study to dissect the specific chemical composition of frankincense oil nor determine its efficacious dosage, since some reports indicated that total frankincense extract is more potent than pure, specific boswellic acids [9]. However, a standard assessment between chemical composition of frankincense oil and its efficacy in tumor suppression will be required in future clinical trials. In addition, frankincense oil was directed added to cell culture media in this study without the inclusion of a carrier; and dose-dependent suppression in cell viability was observed in both J82 and UROtsa cells in the absence of any oil carrier. The absence of carrier eliminated carrier-dependent effects of frankincense resin extract as reported by Chevrier *et al*. [4].

Gene expression analysis was terminated within 3 hours following frankincense oil treatment, since isolated RNA quality and quantity were not sufficient for microarray analysis beyond this time point. We reported a total of 122 up- and 47 down-regulated genes with greater than 2-fold induction or suppression over the period of 3 hours. These findings suggested very specific actions of frankincense oil. The genome-wide gene expression analysis supports patterns of stress, activation of cell cycle arrest, suppression of cell proliferation, and activation of apoptotic signaling in frankincense oil-treated J82 cells within 1 hours of stimulation, and some of these processes were sustained for 3 hours.

Based on the temporal regulation of the genes identified by microarray and bioinformatics analysis, we proposed that frankincense oil induces various death pathways in J82 cells. Waves of transcription factors were regulated by frankincense oil from between 30 min and 3 hours. EGR1 was one of the few genes that were rapidly up-regulated within the first 30 min. Although EGR1 has been shown to be an early gene that is immediately up-regulated in other systems and is correlated with DNA repair [28], the mechanism for elevated EGR1 expression by frankincense oil is unclear. EGR1 has been reported to increase transcription of another transcription factor ATF3 [29]; and levels of ATF3 were increased in our system between 30 and 60 min after frankincense exposure. ATF3 is induced by stresses and can bind to DDIT3 [30], which was up-regulated between 2 and 3 hours in our system. In addition, DDIT3 responds to DNA damage [31], and is responsible for cell cycle arrest [32]. Moreover, using the PAINT webtool [33] to search the TRANSFAC database, we identified EGR1 and ATF3 binding sequences upstream of multiple apoptosis-related genes identified in our system. EGR1 can bind 5'-flanking regions of 5 of the identified apoptosis-related genes (FGFR1, GADD45B, HES1, RHOB, and TRIB1) that were up-regulated between 2 and 3 hours. Binding sites for ATF3 were found in the 5'-flanking regions of 3 apoptosis-related genes (FOSB, GEM, and LAMA5) that were up-regulated between 1 and 2 hours, and 3 additional genes (HSPA1A, ID1, and JUN) between 2 and 3 hours. By similar inference, ATF3 may also account for the expression of 2 down-regulated genes: ING4 (1–2 hours) and ATG5 (2–3 hours). The sequential expression of these identified transcription factors may be ultimately responsible for cell cycle arrest, suppressed cell proliferation, and apoptosis in frankincense oil-treated J82 cells.

Suppressed cell viability and proliferation in frankincense oil-treated J82 cells was also confirmed by elevated expression of genes that are responsible for cell cycle arrest and anti-proliferation. Up-regulated IL8 [34] and CDKN1A [35] have been shown to be responsible for cell cycle arrest and suppressed cell proliferation. IL1A is a negative regulator for cell cycle progression and cell proliferation [36]; and IL6 has been shown to induce growth arrest [37]. However, levels of ING4, a molecule that is a negative regulation of cell proliferation in a human hepatocellular liver carcinoma cell line (HepG2) [38], were up-regulated in frankincense oil-treated cells. ING4 may function differently between J82 cells and HepG2 cells; or ING4 activity is suppressed by a large number of pro-apoptotic molecules in response to frankincense oil.

Frankincense oil up-regulated several pro-apoptotic genes, including CDKN1A [39], DEDD2 [40], NUDT2 [41], SGK, TNFAIP3, and IER3 [42]. Elevated expression (between 0.5 and 2 hours) followed by suppressed expression (2–3 hours) of a cell survival membrane receptor, AXL [43], suggests that cells may try to prolong cell survival following frankincense oil exposure. We also propose that frankincense oil might activate both extrinsic and intrinsic death signaling in J82 cells through stress and death receptors, respectively, to execute apoptosis. Intrinsic death signaling was suggested by up-regulated expression of SSRT1, GADD45B, DDIT3, CDKN1A that have been shown to be required for DNA damage-induced cell cycle arrest [35,39]. Extrinsic death signaling was implicated by the up-regulated expression of DEDD2, which is a death domain receptors and induces apoptosis [40].

Although the bioinformatics and TUNEL analyses reported here suggested that frankincense oil induced apoptosis, rather than necrosis, in J82 cells, frankincense oil did not cause DNA fragmentation, a hallmark of apoptosis, in this bladder cancer cell line. It is possible that DNA fragmentation occurred between 6 and 12 hours post frankincense oil treatment. Alternatively, apoptosis without DNA fragmentation has been reported in several occasions [44-46]; and frankincense oil-induced J82 cell death may fit in this category. The detailed molecular and biological pathways utilized by frankincense oil in inducing bladder cancer cell specific cell death require further investigation.

This study helps to show that frankincense oil may be appropriate as an alternative therapy for bladder cancer. This is the first report demonstrating that frankincense oil can discriminate between bladder cancer cells and normal urothelial cells in a cell culture system, and utilizing microarray technology to identify potential biological pathways activated by frankincense oil. Our results are consistent with a news report that frankincense oil specifically targets malignant melanoma but not normal skin cells in horses . Future studies are required to determine whether frankincense oil has similar effects on other bladder cancer cell lines of varying severity such as RT4, T24, and 5637, followed by *in vivo *studies using bladder cancer animal models. In addition, a standard manufacturing and indication needs to be applied before the commercial frankincense oil can be used as an alternative or complementary therapy for treating bladder cancer.

## Conclusion

Frankincense oil can discriminate bladder cancer cells and normal urothelial cells in culture. The oil suppresses cell survival and induces apoptosis in cultured bladder cancer cells. Based on this preliminary observation, frankincense oil may represent an alternative intravesical therapy for bladder cancer, although more bladder cancer cell lines and animal models need to be used to confirm current observations.

## Competing interests

The authors declare that they have no competing interests.

## Authors' contributions

MBF and JO conducted microarray and bioinformatics analysis. QY, JTA, and MRS performed cell biology and apoptosis analysis of cultured bladder cells. RS, RAA, JCW, KMF, and HKL conceived the idea, designed the experiments, and interpreted the experimental results. All authors contributed to manuscript preparations and approved the final manuscript.

## Pre-publication history

The pre-publication history for this paper can be accessed here:



## Supplementary Material

Additional file 1 **Genes with minimum two-fold increase in adjacent time points. **The data provided a list of all genes whose levels of expression are elevated at least two folds from one time point to the next time point. Click here for file

Additional file 2 **Genes with minimum two-fold decrease in adjacent time points. **The data listed all genes whose levels of expression are suppressed at least two folds from one time point to the next time point. Click here for file

Additional file 3 **Functional groups of frankincense oil-regulated genes in bladder cancer J82 cells. **The data provided the gene ontology classification for all genes that are regulated by frankincense oil. Click here for file
