# Supplementary material for: Frankincense oil derived from Boswellia carteri induces tumor cell specific cytotoxicity
Source: BMC Complement Altern Med. 2009 Mar 18;9:6. doi: 10.1186/1472-6882-9-6 (PMC2664784; doi:10.1186/1472-6882-9-6)
Supplement: Additional file 1 — Genes with minimum two-fold increase in adjacent time points. The data provided a list of all genes whose levels of expression are elevated at least two folds from one time point to the next time point. [file 1472-6882-9-6-S1.doc]

Supplementary Table 1: Genes with minimum two-fold increase in adjacent time points

| SYMBOL | Time (hour) | | | | DEFINITION |
| --- | --- | --- | --- | --- | --- |
|  | 0-0.5 | 0.5-1 | 1-2 | 2-3 |  |
| LOC126295 | 2.65 |  |  |  | Zinc finger protein 57 (ZNF57) |
| SNORD48 | 2.51 |  |  |  | Small nucleolar RNA, C/D box 48 chromosome 6 |
| EGR1 | 5.87 | 2.86 |  |  | Early growth response 1 |
| ZNF234 |  | 2.48 |  |  | Zinc finger protein 234 |
| DNAI2 |  | 2.17 |  |  | Dynein, axonemal, intermediate polypeptide 2 |
| KIRREL |  | 2.08 |  |  | Kin of IRRE like (Drosophila) |
| COPG2 |  | 2.07 |  |  | Coatomer protein complex, subunit gamma 2 |
| FOS |  | 5.39 | 5.05 |  | V-fos FBJ murine osteosarcoma viral oncogene homolog |
| IL8 |  | 4.49 | 4.75 |  | Interleukin 8 |
| ZFP36 |  | 4.49 | 2.44 | 2.47 | Zinc finger protein 36, C3H type, homolog (mouse) |
| FOSB |  | 3.90 | 5.03 | 2.15 | FBJ murine osteosarcoma viral oncogene homolog B |
| ATF3 |  | 3.13 | 5.29 | 2.16 | Activating transcription factor 3, transcript variant 4 |
| CXCL2 |  | 2.60 | 2.43 | 2.18 | Chemokine (C-X-C motif) ligand 2 |
| DUSP5 |  | 2.13 | 2.24 | 2.46 | Dual specificity phosphatase 5 |
| HIST1H4H |  | 2.06 | 3.67 | 2.60 | Histone cluster 1, H4h |
| PHLDA1 |  | 2.71 |  | 2.61 | Pleckstrin homology-like domain, family A, member 1 |
| HIST2H2AA3 |  | 2.35 |  | 2.31 | Histone cluster 2, H2aa3 |
| KLF2 |  | 2.01 |  | 2.32 | Kruppel-like factor 2 (lung) |
| DUSP10 |  |  | 3.40 |  | Dual specificity phosphatase 10, transcript variant 3 |
| MARCH6 |  |  | 2.86 |  | Membrane-associated ring finger (C3HC4) 6 |
| LAMA5 |  |  | 2.80 |  | Laminin, alpha 5 |
| ARHGAP18 |  |  | 2.76 |  | Rho GTPase activating protein 18 |
| TNFAIP3 |  |  | 2.67 |  | Tumor necrosis factor, alpha-induced protein 3 |
| KLF4 |  |  | 2.65 |  | Kruppel-like factor 4 (gut) |
| HIST1H4E |  |  | 2.64 |  | Histone cluster 1, H4e |
| PLXNA3 |  |  | 2.58 |  | Plexin A3 |
| ZC3H12A |  |  | 2.56 |  | Zinc finger CCCH-type containing 12A |
| NEDD9 |  |  | 2.50 |  | Neural precursor cell expressed, developmentally down-regulated 9, transcript variant 1 |
| NPIP |  |  | 2.48 |  | Nuclear pore complex interacting protein |
| SSTR1 |  |  | 2.48 |  | Somatostatin receptor 1 |
| CABIN1 |  |  | 2.45 |  | Calcineurin binding protein 1 |
| KLF5 |  |  | 2.45 |  | Kruppel-like factor 5 (intestinal) |
| NPAT |  |  | 2.30 |  | Nuclear protein, ataxia-telangiectasia locus |
| DLG1 |  |  | 2.30 |  | Discs, large homolog 1 (Drosophila) |
| C12orf11 |  |  | 2.30 |  | Chromosome 12 open reading frame 11 |
| SLC38A2 |  |  | 2.28 |  | Solute carrier family 38, member 2 |
| PLEC1 |  |  | 2.27 |  | Plectin 1, intermediate filament binding protein 500kDa, transcript variant 11 |
| TRIB1 |  |  | 2.27 |  | Tribbles homolog 1 (Drosophila) |
| ZNF654 |  |  | 2.26 |  | Zinc finger protein 654 |
| ZBTB11 |  |  | 2.21 |  | Zinc finger and BTB domain containing 11 |
| PDE4D |  |  | 2.15 |  | Phosphodiesterase 4D, cAMP-specific (phosphodiesterase E3 dunce homolog, Drosophila) |
| NDST2 |  |  | 2.14 |  | N-deacetylase/N-sulfotransferase (heparan glucosaminyl) 2 |
| SGK |  |  | 2.13 |  | Serum/glucocorticoid regulated kinase |
| CELSR3 |  |  | 2.12 |  | Cadherin, EGF LAG seven-pass G-type receptor 3 (flamingo homolog, Drosophila) |
| PSMD10 |  |  | 2.11 |  | Proteasome (prosome, macropain) 26S subunit, non-ATPase, 10, transcript variant 1 |
| ITPR3 |  |  | 2.11 |  | Inositol 1,4,5-triphosphate receptor, type 3 |
| TAOK1 |  |  | 2.10 |  | TAO kinase 1 |
| CLK1 |  |  | 2.09 |  | CDC-like kinase 1, transcript variant 2 |
| PLXNA1 |  |  | 2.08 |  | Plexin A1 |
| C5orf34 |  |  | 2.05 |  | Chromosome 5 open reading frame 34 |
| KIAA0404 |  |  | 2.03 |  | ATG2 autophagy related 2 homolog A (S. cerevisiae) |
| DUSP2 |  |  | 2.01 |  | Dual specificity phosphatase 2 |
| ANKRD27 |  |  | 2.01 |  | Ankyrin repeat domain 27 (VPS9 domain) |
| INT1 |  |  | 2.00 |  | Integrator complex subunit 1, transcript variant 5 (predicted) |
| HIST1H4B |  |  | 5.46 | 2.76 | Histone cluster 1, H4b |
| HIST1H3D |  |  | 2.96 | 2.19 | Histone cluster 1, H3d |
| DNAJB1 |  |  | 2.53 | 2.54 | DnaJ (Hsp40) homolog, subfamily B, member 1 |
| DDIT4 |  |  | 2.49 | 2.08 | DNA-damage-inducible transcript 4 |
| KRT17 |  |  | 2.32 | 2.34 | Keratin 17 |
| ZFAND2A |  |  | 2.14 | 2.29 | Zinc finger, AN1-type domain 2A |
| HIST1H2BF |  |  | 2.04 | 2.37 | Histone cluster 1, H2bf |
| HSPA1A |  |  |  | 42.21 | Heat shock 70kDa protein 1A |
| NUDT2 |  |  |  | 14.05 | Nudix (nucleoside diphosphate linked moiety X)-type motif 2, transcript variant 3 |
| ID1 |  |  |  | 5.80 | Inhibitor of DNA binding 1, dominant negative helix-loop-helix protein, transcript variant 2 |
| CRYAB |  |  |  | 5.36 | Crystallin, alpha B |
| HES1 |  |  |  | 5.18 | Hairy and enhancer of split 1, (Drosophila) |
| LOC440686 |  |  |  | 4.77 | Aimilar to histone H2B histone family |
| LOC202134 |  |  |  | 4.77 | Hypothetical protein LOC202134 |
| GEM |  |  |  | 4.62 | GTP binding protein overexpressed in skeletal muscle, Transcript variant 1 |
| GSTP1 |  |  |  | 4.43 | Glutathione S-transferase pi |
| HIST1H2AM |  |  |  | 3.57 | Histone cluster 1, H2am |
| RN7SK |  |  |  | 3.52 | RNA, 7SK, nuclear on chromosome 6. |
| RCE1 |  |  |  | 3.33 | RCE1 homolog, prenyl protein peptidase (S. cerevisiae), transcript variant 2 |
| RRAD |  |  |  | 3.21 | Ras-related associated with diabetes |
| UBC |  |  |  | 3.14 | Ubiquitin C |
| NOL10 |  |  |  | 3.03 | Nucleolar protein 10 |
| SNF1LK |  |  |  | 2.99 | SNF1-like kinase |
| HIST2H2AC |  |  |  | 2.84 | Histone cluster 2, H2ac |
| UBTF |  |  |  | 2.76 | Upstream binding transcription factor, RNA polymerase I |
| DCI |  |  |  | 2.64 | Dodecenoyl-Coenzyme A delta isomerase (3,2 trans-enoyl-Coenzyme A isomerase), nuclear gene encoding Mitochondrial protein |
| HIST3H2A |  |  |  | 2.63 | Histone cluster 3, H2a |
| IER5L |  |  |  | 2.57 | Immediate early response 5-like |
| TNRC5 |  |  |  | 2.54 | Trinucleotide repeat containing 5, transcript variant 1 |
| TSC22D1 |  |  |  | 2.53 | TSC22 domain family, member 1, transcript variant 2 |
| ZNF682 |  |  |  | 2.52 | Zinc finger protein 682 |
| OASL |  |  |  | 2.46 | 2'-5'-oligoadenylate synthetase-like, transcript variant 1 |
| ABL2 |  |  |  | 2.44 | V-abl Abelson murine leukemia viral oncogene homolog 2, transcript variant a |
| SLC4A5 |  |  |  | 2.37 | Solute carrier family 4, sodium bicarbonate cotransporter, member 5, transcript variant c |
| FLJ46109 |  |  |  | 2.36 | Glutathione S-transferase theta pseudogene on chromosome 22 |
| IL6 |  |  |  | 2.35 | Interleukin 6 (interferon, beta 2) |
| SNAPC1 |  |  |  | 2.31 | Small nuclear RNA activating complex, polypeptide 1, 43kDa |
| TFIP11 |  |  |  | 2.30 | Tuftelin interacting protein 11, transcript variant 1 |
| HIST1H2AC |  |  |  | 2.30 | Histone cluster 1, H2ac |
| CDKN1A |  |  |  | 2.29 | Cyclin-dependent kinase inhibitor 1A (p21, Cip1), transcript variant 2 |
| RIN1 |  |  |  | 2.27 | Ras and Rab interactor 1 |
| IER5 |  |  |  | 2.27 | Immediate early response 5 |
| FLJ90652 |  |  |  | 2.27 | Coiled-coil domain containing 95 |
| OVGP1 |  |  |  | 2.25 | Oviductal glycoprotein 1, 120kDa |
| HMOX1 |  |  |  | 2.25 | Heme oxygenase (decycling) 1 |
| DDIT3 |  |  |  | 2.24 | DNA-damage-inducible transcript 3 |
| RHOB |  |  |  | 2.24 | Ras homolog gene family, member B |
| N4BP2 |  |  |  | 2.22 | Nedd4 binding protein 2 |
| CCL2 |  |  |  | 2.20 | Chemokine (C-C motif) ligand 2 |
| FBXL15 |  |  |  | 2.20 | F-box and leucine-rich repeat protein 15 |
| PPFIBP1 |  |  |  | 2.19 | PTPRF interacting protein, binding protein 1 (liprin beta 1), transcript variant 2 |
| LOC400986 |  |  |  | 2.19 | Protein immuno-reactive with anti-PTH polyclonal antibodies |
| SLC35E1 |  |  |  | 2.19 | Solute carrier family 35, member E1 |
| FGFR1 |  |  |  | 2.18 | Fibroblast growth factor receptor 1 (fms-related tyrosine kinase 2, Pfeiffer syndrome), transcript variant 8 |
| JUN |  |  |  | 2.15 | Jun oncogene |
| MBNL2 |  |  |  | 2.11 | Muscleblind-like 2 (Drosophila), transcript variant 1 |
| H2BFS |  |  |  | 2.10 | H2B histone family, member S |
| DEDD2 |  |  |  | 2.10 | Death effector domain containing 2 |
| FAM46A |  |  |  | 2.08 | Family with sequence similarity 46, member A |
| GADD45B |  |  |  | 2.08 | Growth arrest and DNA-damage-inducible, beta |
| IER3 |  |  |  | 2.05 | Immediate early response 3, transcript variant long |
| FAM40B |  |  |  | 2.05 | Family with sequence similarity 40, member B |
| DENR |  |  |  | 2.05 | Density-regulated protein |
| JUNB |  |  |  | 2.04 | Jun B proto-oncogene |
| ZNF652 |  |  |  | 2.04 | Zinc finger protein 652 |
| IL1A |  |  |  | 2.03 | Interleukin 1, alpha |
| SLCO4A1 |  |  |  | 2.02 | Solute carrier organic anion transporter family, member 4A1 |
| CCL5 |  |  |  | 2.00 | Chemokine (C-C motif) ligand 5 |
