# Supplementary material for: Frankincense oil derived from Boswellia carteri induces tumor cell specific cytotoxicity
Source: BMC Complement Altern Med. 2009 Mar 18;9:6. doi: 10.1186/1472-6882-9-6 (PMC2664784; doi:10.1186/1472-6882-9-6)
Supplement: Additional file 2 — Genes with minimum two-fold decrease in adjacent time points. The data listed all genes whose levels of expression are suppressed at least two folds from one time point to the next time point. [file 1472-6882-9-6-S2.doc]

Supplementary Table 2: Genes with minimum two-fold decrease in adjacent time points

| SYMBOL | Time (hours) | | | | DEFINITION |
| --- | --- | --- | --- | --- | --- |
|  | 0-0.5 | 0.5-1 | 1-2 | 2-3 |  |
| TUBG1 |  | -2.34 |  |  | Tubulin, gamma 1, transcript variant 4 (predicted) |
| VPS11 |  | -2.12 |  |  | Vacuolar protein sorting 11 homolog (S. cerevisiae) |
| POLR2K |  | -2.00 |  |  | Polymerase (RNA) II (DNA directed) polypeptide K, 7.0kDa |
| TNRC5 |  |  | -2.72 |  | Trinucleotide repeat containing 5, transcript variant 1 |
| DNAI2 |  |  | -2.71 |  | Dynein, axonemal, intermediate polypeptide 2 |
| DCI |  |  | -2.61 |  | Dodecenoyl-Coenzyme A delta isomerase (3,2 trans-enoyl-coenzyme A isomerase), nuclear gene encoding mitochondrial protein |
| SNORD36C |  |  | -2.54 |  | Small nucleolar RNA, C/D box 36C on chromosome 9 |
| GABARAP |  |  | -2.34 |  | GABA(A) receptor-associated protein |
| OVGP1 |  |  | -2.22 |  | Oviductal glycoprotein 1, 120kDa (mucin 9, oviductin) |
| NOL12 |  |  | -2.16 |  | Nucleolar protein 12 |
| SURF2 |  |  | -2.12 |  | Surfeit 2 |
| CMTM8 |  |  | -2.10 |  | CKLF-like MARVEL transmembrane domain containing 8 |
| H2AFX |  |  | -2.09 |  | H2A histone family, member X |
| ING4 |  |  | -2.02 |  | Inhibitor of growth family, member 4, transcript variant 2 |
| FKSG24 |  |  | -2.02 |  | Hypothetical protein MGC12972 |
| CABIN1 |  |  |  | -7.98 | Calcineurin binding protein 1 |
| C5orf34 |  |  |  | -3.60 | Chromosome 5 open reading frame 34 |
| TMEM106B |  |  |  | -3.13 | Transmembrane protein 106B |
| IPO7 |  |  |  | -3.05 | Importin 7 |
| LOC723972 |  |  |  | -2.60 | Hepatopoietin PCn127 on chromosome 15 |
| PPP3R1 |  |  |  | -2.59 | Protein phosphatase 3 (formerly 2B), regulatory subunit B, alpha isoform |
| SSTR1 |  |  |  | -2.59 | Somatostatin receptor 1 |
| TAF15 |  |  |  | -2.53 | TAF15 RNA polymerase II, TATA box binding protein (TBP)-Associated factor, 68kDa, transcript variant 2 |
| TPM1 |  |  |  | -2.49 | Tropomyosin 1 (alpha), transcript variant 6 |
| FLG |  |  |  | -2.47 | Filaggrin |
| RAI1 |  |  |  | -2.38 | Retinoic acid induced 1 |
| PLXNA3 |  |  |  | -2.38 | Plexin A3 |
| ZBTB11 |  |  |  | -2.31 | Zinc finger and BTB domain containing 11 |
| RNF123 |  |  |  | -2.28 | Ring finger protein 123 |
| ANKRD27 |  |  |  | -2.24 | Ankyrin repeat domain 27 (VPS9 domain) |
| UTP15 |  |  |  | -2.18 | UTP15, U3 small nucleolar ribonucleoprotein, homolog (S. cerevisiae) |
| AXL |  |  |  | -2.17 | AXL receptor tyrosine kinase, transcript variant 1 |
| APP |  |  |  | -2.16 | Amyloid beta (A4) precursor protein (peptidase nexin-II, Alzheimer disease), transcript variant 3 |
| HDAC4 |  |  |  | -2.16 | Histone deacetylase 4 |
| FLJ12716 |  |  |  | -2.14 | FLJ12716 protein, transcript variant 2 |
| ATP1B2 |  |  |  | -2.14 | ATPase, Na+/K+ transporting, beta 2 polypeptide |
| MAPK8IP3 |  |  |  | -2.11 | Mitogen-activated protein kinase 8 interacting protein 3, Transcript variant 2 |
| NDST2 |  |  |  | -2.09 | N-deacetylase/N-sulfotransferase (heparan glucosaminyl) 2 |
| GOPC |  |  |  | -2.08 | golgi associated PDZ and coiled-coil motif containing, Transcript variant 1 |
| PRPF8 |  |  |  | -2.07 | PRP8 pre-mRNA processing factor 8 homolog (yeast) |
| CROP |  |  |  | -2.06 | cisplatin resistance-associated overexpressed protein, Transcript variant 1 |
| ATG5 |  |  |  | -2.05 | ATG5 autophagy related 5 homolog (S. cerevisiae) |
| MTMR6 |  |  |  | -2.03 | Myotubularin related protein 6 |
| TUBD1 |  |  |  | -2.03 | Tubulin, delta 1 |
| KIAA0404 |  |  |  | -2.02 | ATG2 autophagy related 2 homolog A (S. cerevisiae) |
| DEPDC1B |  |  |  | -2.00 | DEP domain containing 1B |
| ABCA3 |  |  |  | -2.00 | ATP-binding cassette, sub-family A, member 3 |
