# Supplementary material for: Frankincense oil derived from Boswellia carteri induces tumor cell specific cytotoxicity
Source: BMC Complement Altern Med. 2009 Mar 18;9:6. doi: 10.1186/1472-6882-9-6 (PMC2664784; doi:10.1186/1472-6882-9-6)
Supplement: Additional file 3 — Functional groups of frankincense oil-regulated genes in bladder cancer J82 cells. The data provided the gene ontology classification for all genes that are regulated by frankincense oil. [file 1472-6882-9-6-S3.doc]

Supplementary Table 3. Functional groups of frankincense oil-regulated genes in bladder cancer J82 cells

| **Function** | **Gene Symbol** | **Description** |
| --- | --- | --- |
| Cytokines |  |  |
|  | CCL2 | chemokine (C-C motif) ligand 2 |
|  | CCL5 | chemokine (C-C motif) ligand 5 |
|  | CMTM8 | CKLF-like MARVEL transmembrane domain containing 8 |
|  | CXCL2 | chemokine (C-X-C motif) ligand 2 |
|  | IL1A | interleukin 1, alpha |
|  | IL6 | interleukin 6 (interferon, beta 2) |
|  | IL8 | interleukin 8 |
| Enzymes - kinases | |  |
|  | ABL2 | v-abl Abelson murine leukemia viral oncogene homolog 2 (arg, Abelson-related gene) |
|  | AXL | AXL receptor tyrosine kinase |
|  | CDKN1A | cyclin-dependent kinase inhibitor 1A (p21, Cip1) |
|  | CLK1 | CDC-like kinase 1 |
|  | DLG1 | discs, large homolog 1 (Drosophila) |
|  | FGFR1 | fibroblast growth factor receptor 1 (fms-related tyrosine kinase 2, Pfeiffer syndrome) |
|  | PSTK | phosphoseryl-tRNA kinase |
|  | SGK1 | serum/glucocorticoid regulated kinase 1 |
|  | SNF1LK | SNF1-like kinase |
|  | TAOK1 | TAO kinase 1 |
|  | TRIB1 | tribbles homolog 1 (Drosophila) |
| Enzymes - peptidases | |  |
|  | RCE1 | RCE1 homolog, prenyl protein peptidase (S. cerevisiae) |
| Enzymes - phosphatases | |  |
|  | DUSP10 | dual specificity phosphatase 10 |
|  | DUSP2 | dual specificity phosphatase 2 |
|  | DUSP5 | dual specificity phosphatase 5 |
|  | MTMR6 | myotubularin related protein 6 |
|  | NUDT2 | nudix (nucleoside diphosphate linked moiety X)-type motif 2 |
|  | PPP3R1 | protein phosphatase 3 (formerly 2B), regulatory subunit B, alpha isoform |
|  | PTPN23 | protein tyrosine phosphatase, non-receptor type 23 |
| Enzymes - other | |  |
|  | DCI | dodecenoyl-Coenzyme A delta isomerase (3,2 trans-enoyl-Coenzyme A isomerase) |
|  | FBXO9 | F-box protein 9 |
|  | GEM | GTP binding protein overexpressed in skeletal muscle |
|  | GSTM2 | glutathione S-transferase M2 (muscle) |
|  | GSTM4 | glutathione S-transferase M4 |
|  | GSTP1 | glutathione S-transferase pi |
|  | HMOX1 | heme oxygenase (decycling) 1 |
|  | NDST2 | N-deacetylase/N-sulfotransferase (heparan glucosaminyl) 2 |
|  | OASL | 2'-5'-oligoadenylate synthetase-like |
|  | OVGP1 | oviductal glycoprotein 1, 120kDa (mucin 9, oviductin) |
|  | PDE4D | phosphodiesterase 4D, cAMP-specific (phosphodiesterase E3 dunce homolog, Drosophila) |
|  | POLR2K | polymerase (RNA) II (DNA directed) polypeptide K, 7.0kDa |
|  | RHOB | ras homolog gene family, member B |
|  | RRAD | Ras-related associated with diabetes |
|  | ZDHHC2 | zinc finger, DHHC-type containing 2 |
| Membrane Receptors | |  |
|  | PLAUR | plasminogen activator, urokinase receptor |
|  | PLXNA1 | plexin A1 |
|  | PLXNA3 | plexin A3 |
|  | SSTR1 | somatostatin receptor 1 |
| Ion Channels | |  |
|  | ITPR3 | inositol 1,4,5-triphosphate receptor, type 3 |
| Molecular Transport | |  |
|  | ABCA3 | ATP-binding cassette, sub-family A (ABC1), member 3 |
|  | ATP1B2 | ATPase, Na+/K+ transporting, beta 2 polypeptide |
|  | COPG2 | coatomer protein complex, subunit gamma 2 |
|  | GABARAP | GABA(A) receptor-associated protein |
|  | GOPC | golgi associated PDZ and coiled-coil motif containing |
|  | IPO7 | importin 7 |
|  | SEC24A | SEC24 related gene family, member A (S. cerevisiae) |
|  | SLC38A2 | solute carrier family 38, member 2 |
|  | SLCO4A1 | solute carrier organic anion transporter family, member 4A1 |
|  | VPS11 | vacuolar protein sorting 11 homolog (S. cerevisiae) |
| Other |  |  |
|  | APP | amyloid beta (A4) precursor protein (peptidase nexin-II, Alzheimer disease) |
|  | ARHGAP18 | Rho GTPase activating protein 18 |
|  | ARHGEF11 | Rho guanine nucleotide exchange factor (GEF) 11 |
|  | ATG5 | ATG5 autophagy related 5 homolog (S. cerevisiae) |
|  | C12ORF11 | chromosome 12 open reading frame 11 |
|  | C5ORF34 | chromosome 5 open reading frame 34 |
|  | CGN | cingulin |
|  | CHMP6 | chromatin modifying protein 6 |
|  | CRYAB | crystallin, alpha B |
|  | DDIT4 | DNA-damage-inducible transcript 4 |
|  | DEDD2 | death effector domain containing 2 |
|  | DENR | density-regulated protein |
|  | DNAI2 | dynein, axonemal, intermediate chain 2 |
|  | DNAJB1 | DnaJ (Hsp40) homolog, subfamily B, member 1 |
|  | FAM46A | family with sequence similarity 46, member A |
|  | FLG | filaggrin |
|  | GADD45B | growth arrest and DNA-damage-inducible, beta |
|  | H2AFX | H2A histone family, member X |
|  | HIST1H2AC | histone cluster 1, H2ac |
|  | HIST1H2AM | histone cluster 1, H2am |
|  | HIST1H3D | histone cluster 1, H3d |
|  | HIST1H4H | histone cluster 1, H4h |
|  | HIST2H2AA3 | histone cluster 2, H2aa3 |
|  | HSPA1A | heat shock 70kDa protein 1A |
|  | IER3 | immediate early response 3 |
|  | IER5 | immediate early response 5 |
|  | IER5L | immediate early response 5-like |
|  | ING4 | inhibitor of growth family, member 4 |
|  | KIRREL | kin of IRRE like (Drosophila) |
|  | KRT17 | keratin 17 |
|  | LAMA5 | laminin, alpha 5 |
|  | MAD2L1 | MAD2 mitotic arrest deficient-like 1 (yeast) |
|  | MAPK8IP3 | mitogen-activated protein kinase 8 interacting protein 3 |
|  | MBNL2 | muscleblind-like 2 (Drosophila) |
|  | NEDD9 | neural precursor cell expressed, developmentally down-regulated 9 |
|  | NPAT | nuclear protein, ataxia-telangiectasia locus |
|  | NPIP | nuclear pore complex interacting protein |
|  | PHLDA1 | pleckstrin homology-like domain, family A, member 1 |
|  | PLEC1 | plectin 1, intermediate filament binding protein 500kDa |
|  | PPFIBP1 | PTPRF interacting protein, binding protein 1 (liprin beta 1) |
|  | PRPF8 | PRP8 pre-mRNA processing factor 8 homolog (S. cerevisiae) |
|  | RIN1 | Ras and Rab interactor 1 |
|  | RN7SK | RNA, 7SK, nuclear |
|  | RNF123 | ring finger protein 123 |
|  | SH3BGRL2 | SH3 domain binding glutamic acid-rich protein like 2 |
|  | SNAPC1 | small nuclear RNA activating complex, polypeptide 1, 43kDa |
|  | TFIP11 | tuftelin interacting protein 11 |
|  | TNFAIP3 | tumor necrosis factor, alpha-induced protein 3 |
|  | TPM1 | tropomyosin 1 (alpha) |
|  | TRO | trophinin |
|  | TUBD1 | tubulin, delta 1 |
|  | TUBG1 | tubulin, gamma 1 |
|  | UBC | ubiquitin C |
|  | WNT1 | wingless-type MMTV integration site family, member 1 |
|  | ZBTB11 | zinc finger and BTB domain containing 11 |
|  | ZC3H12A | zinc finger CCCH-type containing 12A |
